# Anticoagulant therapy likely increases risk of bleeding in *Gynura segetum*-induced hepatic sinus obstruction syndrome

**DOI:** 10.1097/MD.0000000000035914

**Published:** 2024-02-09

**Authors:** Youwen Tan, Xingbei Zhou

**Affiliations:** a Department of Hepatology, The Third Hospital of Zhenjiang Affiliated Jiangsu University, Daijiamen, Runzhou Distinct, Zhenjiang, China.

**Keywords:** anticoagulant therapy, clinical features, hemorrhage, hepatic sinusoidal obstruction syndrome, Tusanqi

## Abstract

This study aimed to analyze the clinical characteristics of *Gynura segetum* (Tusanqi)-induced hepatic sinusoidal obstruction syndrome (HSOS) and the benefits and risks of anticoagulant therapy for Tusanqi-induced HSOS. This was a retrospective analysis of 49 patients with Tusanqi-induced HSOS who were treated with anticoagulation or standard therapy between July 2006 and December 2022. Clinical manifestations included abdominal pain (n = 47) and peritoneal or pleural effusion (n = 46); 2 patients died. Nineteen patients requested standard medical treatment, while 30 were treated with anticoagulants. HSOS resolved within 6 months in 22 patients but did not resolve in 27 patients. The resolution rate was higher in the anticoagulant than standard treatment group (*P* = .037). Logistic regression analysis revealed that a history of chronic liver disease or treatment increased the risk of poor outcomes. Bleeding complications occurred in 6 patients in the anticoagulant treatment group. Early diagnosis and anticoagulant treatment are beneficial for rapid recovery after Tusanqi-induced HSOS. However, anticoagulant treatment is associated with the risk of multisite bleeding.

## 1. Introduction

Hepatic sinusoidal obstruction syndrome (HSOS) is characterized by edema, necrosis, and abscission of endothelial cells in the hepatic sinusoids, hepatic venules, and interlobular veins resulting in microthrombosis, intrahepatic congestion, functional liver injury, and acute portal hypertension. Tusanqi has become the main cause of HSOS in China because it contains pyrrolidine alkaloids (PAs) that can damage hepatic sinusoidal endothelial cells and cause HSOS.^[1–3]^ There is currently no specific treatment for HSOS. Once the clinical diagnosis is made, Chinese herbal medicines containing PAs should be discontinued. Routine treatments, such as supportive measures, diuresis, and management aimed at improving microcirculation, may effectively improve the prognosis of mild HSOS. Among patients who respond poorly to medical treatment, anticoagulants are an effective treatment option. Some researchers have reported the use of unfractionated or low-molecular-weight heparin for the treatment of Tusanqi-induced HSOS with success rates of 70% to 88.9%.^[4–7]^ Anticoagulant therapy is associated with serious complications such as bleeding, which renders its use controversial. Therefore, here we retrospectively analyzed the clinical characteristics of 49 patients with Tusanqi-induced HSOS as well as the benefits and risks associated with anticoagulant therapy.

## 2. Methods

### 2.1. Objectives and methodology

Patient selection and diagnostic criteria: The study included 49 patients (29 men, 20 women; mean age, 64.46 ± 8.68 years) with abdominal distension, abdominal pain, and abnormal liver function after ingesting Tusanqi who were admitted to Zhenjiang Third Hospital Affiliated to Jiangsu University between July 2006 and December 2022. The duration between ingestion and disease onset was 1 to 2 months in 47 patients. All patients received standard care, including supportive treatment and measures aimed at improving their microcirculation and promoting diuresis. In the anticoagulant treatment group, anticoagulant treatment was added to standard medical treatment (low-molecular-weight heparin and/or warfarin, n = 27; rivaroxaban, n = 3). Seventeen patients had a history of chronic liver disease, 9 had alcoholic liver disease, 4 had chronic hepatitis B, one had chronic hepatitis C, 2 had hepatic schistosomiasis, and one had autoimmune liver disease.

Disease resolution was identified when a patient’s clinical symptoms had remitted, biochemical indices were normal, and imaging examinations revealed normal findings. Unresolved disease was identified when a patient’s clinical symptoms had not remitted, the biochemical indices were abnormal, and liver imaging showed evidence of lesions caused by Tusanqi after 6 months. The diagnosis of Tusanqi-induced HSOS was based on the Baltimore and Seattle standards.^[8]^ The course of Tusanqi-induced HSOS can be divided into acute, subacute, and chronic phases.^[9,10]^

The study was approved by the Medical Ethics Committee of the Third Hospital of Zhenjiang Affiliated Jiangsu University (no. 202215), which waived the requirement for written informed consent due to the study’s retrospective design. This study was conducted in accordance with the principles of the Declaration of Helsinki.

### 2.2. Observation indexes

Data on the following general demographic indices were retrospectively collected: sex, age, history of hepatic disease, method of ingesting Tusanqi (as decoction, powder, or wine), duration of ingestion, and duration from Tusanqi ingestion to disease onset. The recorded biochemical indices included baseline and most abnormal values of total bilirubin (TBIL), alanine aminotransferase, alkaline phosphatase, albumin, platelets (PLT), international normalized ratio, and d-dimer. Abdominal computed tomography was used to assess extrahepatic manifestations, such as ascites and abnormalities of the gallbladder, spleen, and blood vessels (collateral vein shunt). The arterial, venous, balance, and delayed phases were evaluated using plain and enhanced hepatic computed tomography to assess intrahepatic manifestations. The findings were classified as mild (mild heterogeneous hypoattenuation, hepatic vein narrowing, unclear view of one main hepatic vein, and small pleural effusion and ascites), moderate (moderate heterogeneous hypoattenuation, different enhancement, hepatic vein narrowing, unclear view of 2 main hepatic veins, and moderate pleural effusion and ascites), or severe (marked heterogeneous hypoattenuation, unrecognizable hepatic veins, and large pleural effusion and ascites).

### 2.3. Statistical analysis

The statistical analysis was performed using SPSS v. 23.0 for Windows (SPSS Inc., Chicago, IL). The data are expressed as mean ± standard deviation. Two means were tested using Student *t*-test, while multiple means were analyzed using one-way analysis of variance. The chi-squared test was used to examine nominal data. A multivariate (binary) logistic regression analysis was performed to analyze the risk factors.

## 3. Results

### 3.1. Overall clinical characteristics and outcomes

The main clinical manifestations included abdominal pain (n = 47), peritoneal or pleural effusion (n = 46), simple peritoneal effusion (n = 40), and effusions involving the chest and the abdominal cavity (n = 10). Subacute liver failure was diagnosed in 6 patients; 2 patients died. Nineteen patients requested standard care, while 30 received combination anticoagulant treatment for 1 to 3 months.

### 3.2. Clinical characteristics of patients with versus without disease resolution

The disease resolved in 22 patients within 6 months of treatment but not in 27 patients. Sex, age, mode of administration, presence of pleural effusions and ascites, main biochemical indices, PLT count, and coagulation indices did not differ significantly between groups (*P* > .05). The resolution rate was lower in patients with versus without a history of chronic liver disease. Moreover, the resolution rate was higher among patients receiving anticoagulant therapy versus standard treatment (*P* < .05). The highest TBIL level in the unresolved disease group was significantly higher than that in the anticoagulant treatment group (*P* < .05). These differences were likely related to more serious imaging manifestations in the unresolved versus resolved disease group (*P* < .05; Table 1). Disease resolution and unresolved disease were considered binary dependent variables. Treatment mode, history of chronic liver disease, peak TBIL level, imaging characteristics, and presence of pleural effusion and ascites were independent variables in the univariate analysis (*P* < .1). A stepwise regression analysis showed that a history of chronic liver disease and treatment mode were risk factors for unresolved disease (Table 2).

**Table 1:** Comparison of clinical characteristics between cured group and noncured group.

Parameter	Cured group (n = 22)	No cured group (n = 27)	Statistical value	*P*
Sex				
Woman	11 (50.0%)	9 (33.3%)	1.394	.238
Man	11 (50.0%)	18 (66.7%)		
Age(year)	67.27 ± 7.57	62.18 ± 8.98	1.526	.233
<65	8 (36.4%)	14 (51.9%)	1.175	.278
≥65	14 (83.6%)	13 (48.1%)		
Way of taking				
Soak wine	3 (13.6%)	7 (25.9%)	1.127	.288
Water decoction	19 (86.4%)	20 (74.1%)		
Duration time (d)	44.01 ± 32.97	49.21 ± 64.22	1.694	.199
History of chronic liver disease				
No	19 (86.4%)	13 (48.1%)	6.566	.008
Yes	3 (13.6%)	14 (51.9%)		
Treatment				
Traditional	5 (22.7%)	14 (51.9%)	4.331	.037
Combined anticoagulation	17 (77.3%)	13 (48.1%)		
Biochemical index (baseline)				
TBIL	47.37 ± 36.48	49.63 ± 40.47	0.002	.96
ALT	187.64 ± 315.75	245.66 ± 232.64	0.009	.926
ALP	173.64 ± 198.63	160.35 ± 98.07	0.269	.607
ALB	34.94 ± 3.46	34.03 ± 3.86	0.803	.375
Biochemical index (Peak)				
TBIL	36.73 ± 40.12	122.63 ± 177.01	9.037	.004
ALT	84.82 ± 81.86	116.12 ± 155.53	2.761	.103
ALP	123.82 ± 39.52	132.84 ± 43.76	0.006	.94
ALB	31.03 ± 3.35	32.81 ± 3.64	0.151	.699
Coagulation index (baseline)				
PT	14.56 ± 3.29	15.53 ± 4.83	0.352	.241
INR	1.25 ± 0.86	1.33 ± 1.01	0.012	.742
D-er	1443.52 ± 1202.58	1908.64 ± 1299.63	0.317	.576
PLT	92.36 ± 36.77	87.07 ± 31.37	1.58	.215
Imaging features				
Mlid	13 (59.1%)	8 (29.6%)	8.974	.011
Moderate	9 (40.1%)	11 (40.7%)		
Severe	0 (0%)	8 (29.6%)		
Pleural effusion and ascites				
Mlid or no	13 (59.1%)	7 (25.9%)	5.706	.058
Moderate	6 (27.3%)	15 (55.6%)		
Severe	3 (13.6%)	5 (18.5%)		
Clinical phase				
Acute/subacute	22 (90.9%)	24 (88.9%)	0.054	.816
Chronic	2 (9.1%)	3 (11.1%)		

ALP = alkaline phosphatase, ALT = alanine aminotransferase, D-er = D-dimer, INR = internationalization ratio, PLT = platelet, PT = prothrombin time, TBIL = total bilirubin.

**Table 2:** Logistic regression analysis of the variables of the cured and the noncured group.

Variables	β	Wald	Exp (95%CI)	*P*
History of chronic liver disease	2.434	4.232	11.4 (1.165–24.395)	.023
Treatment	1.274	3.4	3.574 (0.550–9.815)	.045
TBIL (peak)	0.009	1.887	0.991 (0.977–1.004)	.416
Pleural effusion and ascites		1.025		.599
Imaging features		1.675		.433

TBIL: total bilirubin.

### 3.3. Clinical characteristics of standard versus anticoagulant treatment groups

**Figure 1.**
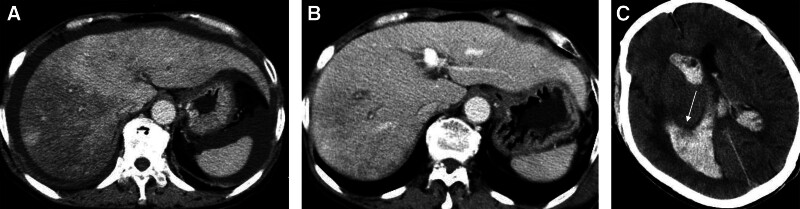
A 52-year-old woman with abdominal pain and distension after using a water-based decoction of Tusanqi for 20 days (A). After 20 days of treatment with low-molecular-weight heparin and warfarin, the liver’s density was uniform, the ascites remitted, and the hepatic veins were clearly visible (B). After she suddenly lost consciousness, computed tomography revealed an intraventricular hemorrhage (C, arrow).

Sex, age, mode of administration, history of chronic liver disease, imaging characteristics, presence of pleural effusions and ascites, main biochemical indices, PLT count, and coagulation indices did not differ significantly between groups (*P* > .05). The disease resolution rate was higher in the anticoagulant than standard treatment group. Regarding discharge indicators, the anticoagulant treatment group fared better in terms of TBIL level and imaging changes than the standard treatment group (*P* < .05). Six patients who underwent transjugular intrahepatic portosystemic shunt placement were assigned to the anticoagulant therapy group. There were 6 cases of bleeding complications in the anticoagulant treatment group (one of gastrointestinal bleeding, 2 of duodenal ulcer bleeding, one of hematuria, one of intracranial bleeding (Fig. 1), and one of massive subcutaneous fascial bleeding) versus only one case of gastrointestinal bleeding in the standard treatment group. Despite not reaching statistical significance (*x*^2^ = 2.06; *P* = .15), multisite bleeding was related to the anticoagulant treatment (Table 3).

**Table 3:** Comparison of clinical characteristics between traditional treatment group and combined anticoagulation group.

Parameter	Traditional treatment group (n = 19)	Combined anticoagulation group (n = 30)	Statistical value	*P*
Sex				
Woman	5 (26.3%)	15 (50%)	2.701	.1
Man	14 (73.7%)	15 (50%)		
Age(year)	63.31 ± 9.05	65.2 ± 8.51	0.005	.951
<65	11 (52.9%)	11 (36.7%)	2.119	.145
≥65	8 (47.1%)	19 (63.3%)		
Way of taking				
Soak wine	5 (26.3%)	5 (16.7%)	0.667	.414
Water decoction	14 (73.7%)	25 (83.3%)		
Duration time (d)	45.15 ± 38.56	46.93 ± 57.56	0.084	.773
History of chronic liver disease				
No	11 (57.9%)	22 (73.3%)	1.261	.261
Yes	8 (42.1%)	8 (26.7%)		
Cured				
No	14 (73.7%)	13 (43.3%)	4.331	.037
Yes	5 (26.3%)	17 (56.7%)		
Biochemical index (baseline)				
TBIL	48.28 ± 25.26	48.44 ± 65.26	0.544	.464
ALT	285.53 ± 314.25	178.03 ± 236.68	3.901	.054
ALP	158.37 ± 76.84	172.52 ± 182.75	1.555	.219
ALB	33.86 ± 3.14	34.81 ± 3.96	0.215	.645
Biochemical index (discharged)				
TBIL	44.24 ± 78.32	25.54 ± 52.75	6.374	.005
ALT	36.25 ± 67.24	32.85 ± 55.64	1.085	.321
ALP	78.45 ± 57.34	52.84 ± 55.73	0.306	.294
ALB	34.65 ± 5.36	35.64 ± 5.64	0.174	.663
Coagulation index (baseline)				
PT	15.37 ± 4.74	16.21 ± 5.53	0.373	.296
INR	1.15 ± 0.63	1.35 ± 0.53	0.332	.221
D-er	1658.43 ± 1197.45	1723.64 ± 1262.53	0.014	.907
PLT	89.53 ± 42.14	93.23 ± 45.27	1.158	.154
Imaging features (baseline)				
Mlid	10 (52.6%)	11 (36.7%)	2.715	.257
Moderate	5 (26.3%)	15 (50%)		
Severe	4 (21.1%)	4 (13.3%)		
Imaging features (discharged)				
Normal	10 (52.6%)	24 (80%)	4.102	.043
Abnormal	9 (47.4%)	6 (20%)		
Pleural effusion and ascites (baseline)				
Mlid	9 (47.4%)	11 (36.7%)	0.97	.616
Moderate	8 (42.1%)	13 (43.3%)		
Severe	2 (10.5%)	6 (20%)		
Pleural effusion and ascites (discharged)				
Normal	11 (73.7%)	22 (76.7%)	1.261	.261
Abnormal	8 (5.1%)	8 (6.7%)		
Bleeding complications				
No	18 (94.7%)	24 (80%)	2.063	.151
Yes	1 (5.3%)	6 (20%)		
TIPS				
No	19 (100%)	24 (80%)	4.242	.039
Yes	0 (0%)	6 (20%)		

ALP = alkaline phosphatase, ALT = alanine aminotransferase, D-er = D-dimer, INR = internationalization ratio, PLT = platelet, PT = prothrombin time, TBIL = total bilirubin, TIPS = transjugular intrahepatic portosystemic shunt.

## 4. Discussion

Tusanqi contains PAs that can damage hepatic sinusoidal endothelial cells, resulting in HSOS.^[11,12]^ In our recent eight-case series of 955 cases of HSOS caused by Tusanqi,^[8]^ 98% to 100% had ascites, 64.8% to 92% had hepatomegaly, and 98.3% to 100% had abdominal pain.

A multicenter study of 117 cases of PA-induced HSOS in China^[10]^ reported 1-, 3-, and 36-month survival rates of 89.71%, 72.60%, and 69.19%, respectively. The early diagnosis and timely treatment of HSOS are the keys to improving survival rates. In another study of 64 cases of PA-induced HSOS from 8 centers in China,^[13]^ the misdiagnosis rate reached 92.18% (59/64), while the 6-month mortality rate was as high as 78%. Forty-four patients in our center were correctly diagnosed and treated in the acute phase, while only 5 patients were diagnosed in the chronic phase, which was the main reason for the 2 deaths in our cohort.

Anticoagulation is the primary treatment for HSOS; however, it does not improve or prevent hematopoietic stem cell therapy-induced HSOS.^[14]^ A systematic review of 2782 patients with HSOS found that anticoagulation therapy may help prevent venous occlusive diseases.^[15]^ In a study of patients with PA-induced HSOS,^[16]^ among the 28 patients who did not receive anticoagulation, 9 (32.1%) were cured and 19 (67.9%) died; among the 75 patients receiving anticoagulation, 49 (65.3%) died, and 26 (34.7%) were cured. The cure rate was higher in the anticoagulant than standard treatment group (*P* = .004).

Another study examined 249 patients with PA-induced HSOS from multiple centers in China^[7]^; among them, 151 received anticoagulant therapy and 98 received supportive therapy. The cumulative complete remission rate was higher in the anticoagulant versus supportive therapy group (60.9% vs 36.7%; *P* < .0001). The cumulative mortality rate in the anticoagulant therapy group was 12.9% (*P* < .0001). There was no significant intergroup difference in the incidence of bleeding events (*P* = .674). Similarly, in our cohort, the cure rate was higher in the anticoagulant versus standard treatment group.

This cohort study has several limitations. First, this was a retrospective single-center study with a small sample size. However, Tusanqi-induced HSOS is an unexpectedly rare disease, making it difficult to conduct a prospective randomized controlled study. Second, the statistical analysis did not show that anticoagulation therapy increased the risk of bleeding. However, it should be noted that the occurrence of bleeding at multiple sites in the anticoagulant group can clearly be attributed to the anticoagulant therapy despite no statistically significant difference due to the small sample size.

In conclusion, here we retrospectively observed that Tusanqi-induced HSOS, an early correct diagnosis, and early anticoagulation therapy were conducive to rapid recovery from the disease, which was also the reason for the high cure rate and extremely low mortality rate among this cohort. However, anticoagulation can induce multisite bleeding, and its benefits and risks require testing in larger samples. This is also the reason for the high cure rate and very low mortality rate of our cohort. However, anticoagulant treatment is associated with a risk of multisite bleeding, and the benefits and risks of anticoagulation for HSOS require confirmation in future studies with larger sample sizes.

## Author contributions

**Conceptualization:** Youwen Tan.

**Data curation:** Xingbei Zhou.

**Funding acquisition:** Youwen Tan.

**Investigation:** Youwen Tan.

**Writing – original draft:** Youwen Tan.

**Writing – review & editing:** Youwen Tan.
